# Complete genome sequence of *Pseudomonas spelaei* strain PML3(3), an effective mineral-weathering bacterial strain isolated from the oak*–Scleroderma citrinum* ectomycorrhizosphere

**DOI:** 10.1128/mra.00247-26

**Published:** 2026-04-20

**Authors:** S. Bouche, C. Oger-Desfeux, E. Morin, F.M. Martin, P. Oger, S. Uroz

**Affiliations:** 1 UMR 1136 IAM, Université de Lorraine, INRAE 137665, Nancy, France; 2 INRAE, UR1138 « Biogéochimie des Écosystèmes Forestiers », Champenoux, France; 3 Université Claude Bernard Lyon1, PRABI-AMSB, FR3728 BioEEnViS, Villeurbanne, France; 4 CNRS UMR5240, INSA Lyon, Université Claude Bernard Lyon1, Villeurbanne, France; DOE Joint Genome Institute, Berkeley, California, USA

**Keywords:** *Pseudomonas spelaei*, mycorrhizosphere, acidic forest soil, mineral weathering

## Abstract

We present the complete genome sequence of *Pseudomonas spelaei* strain PML3(3), isolated from the oak*–Scleroderma citrinum* ectomycorrhizosphere in acidic forest soil at the ecosystem research site of Breuil-Chenue, France. This genome encodes functions related to adaptation to nutrient-poor conditions, competition with other microorganisms, and plant growth promotion.

## ANNOUNCEMENT

More than 300 validly published *Pseudomonas* species are currently described, and this number continues to grow, particularly in soil (1–7). Their enrichment in the (mycor)rhizosphere of various plants raises the question of their roles in plant growth promotion and fungal interactions (2, 3, 8–11). The characterization of their physiology and genomes revealed a functional versatility (2–5) and, for some of them, the ability to promote the growth of mycorrhizal fungi (10). Common in agricultural soils, *Pseudomonas* are relatively rare in acidic forest soils (12–16). This strain was isolated in October 2004 from 20 mg of oak (*Quercus petraea*)*–S. citrinum* ectomycorrhizae. This sample was suspended in 1 mL sterile water and serially diluted on 1/10 tryptic soy agar medium. The strain was cryo-preserved in 40% glycerol (17, 18). Strain PML3(3) was retained for in-depth analysis due to its effectiveness at weathering (18).

Genomic DNA for sequencing was extracted from a 2-day, late exponential phase culture grown at 25°C in AB mannitol medium, as described in Pospiech and Neumann (19). DNA concentration was 1,500 ng/µL (final volume: 500 µL). Nanopore sequencing was done on a MinION device with a flow cell (FLO-MIN106) with the Rapid Sequencing Kit (SQK-RAD003) and using 400 ng of DNA. Illumina sequencing was done on an Illumina MiSeq (GenoScreen, Lille, France) (2 × 150 bp paired reads). The library was prepared using a 117 ng/µL DNA dilution and the Nextera XT DNA library preparation kit (Illumina).

Default parameters were used at all steps except where specified.

Base calling was performed using MinKNOW v1.6 and Albacore v2.0.1, yielding 16,303 long reads (only filtered for quality *q* > 7). Illumina short reads were trimmed and filtered by GenoScreen using sickle v1.33 (20), yielding 1,384,397 read pairs, quality controlled using FastQC v0.11.9 (21). Trimmed short reads (60×) and long reads (34×) were assembled using Unicycler v0.4.8 (22) into a single circular chromosome sequence, rotated to *dnaA*. Assembly was carefully inspected by visualizing the alignment of short and long reads onto the assembly using Minimap2 v2.28 (23), Samtools-1.19 (24), and IGV 2.16.2 (25). Genome completeness was evaluated using BUSCO v6.0.0 (26) with the *Pseudomonas*_odb12 data set.

Gene prediction and annotation were done using the MAGE platform (27). The tool tRNA-scan-SE v2.0.12 (28) was used for tRNA prediction, and Barrnap v0.9 (29) was used for rRNA prediction. The complete statistics of the draft genome can be found in Table 1.

**TABLE 1:** Genomic features of *Pseudomonas* strain PML3(3)

Strain PML3(3)	Illumina	Nanopore	Genome assembly
SRA/GenBank accession no.	SRR37349083	SRX32262954	JBUQCZ000000000.1
Filtered reads	1,384,397 pairs	16,303	
Read N50		30,705	
Coverage	60×	34×	
BUSCO score			97.6% (*Pseudomonas* markers)
Chromosome length (bp)			6,603,948
Average GC%			59.31
Genes			6,217
Predicted coding genes			6,090
tRNA			66
5S rRNA gene			5
16S rRNA gene			5
23S rRNA gene			5

Digital DNA–DNA hybridization (30) values with the closest type strains ranged from 97.6% with *Pseudomonas spelaei* (LMG27931^T^) (31), 80.8% with *Pseudomonas karstica* (LMG27930^T^), and 50.8% with *Pseudomonas yamanorum* (LMG 27247^T^), clearly identifying the strain as *P. spelaei*.

Genes known to confer high mineral weathering effectiveness in *Pseudomonas*, such as *pqq* genes encoding the pyrroloquinoline quinone (PQQ) cofactor, a *gcd* gene encoding a PQQ-dependent glucose dehydrogenase, a *gad* gene encoding a FAD-dependent gluconate dehydrogenase (32), were identified. Genes involved in the synthesis of the siderophores pyoverdine, pseudomonine, and phenazine were also detected (33). These findings suggest that PML3(3) is well equipped to weather minerals, to inhibit fungal and bacterial growth, and to promote plant growth.

## Data Availability

The Illumina and Nanopore raw sequences are available under accession nos. SRR37349083 and SRX32262954, and the whole genome assembly is available under the accession no. JBUQCZ000000000

## References

[1] Udaondo Z, Ramos JL, Abram K. 2024. Unraveling the genomic diversity of the Pseudomonas putida group: exploring taxonomy, core pangenome, and antibiotic resistance mechanisms. FEMS Microbiol Rev 48:fuae025. doi:10.1093/femsre/fuae025 39390673 PMC11585281

[2] Ghirardi S, Dessaint F, Mazurier S, Corberand T, Raaijmakers JM, Meyer J-M, Dessaux Y, Lemanceau P. 2012. Identification of traits shared by rhizosphere-competent strains of fluorescent pseudomonads. Microb Ecol 64:725–737. doi:10.1007/s00248-012-0065-3 22576821

[3] Frey-Klett P, Chavatte M, Clausse M-L, Courrier S, Le Roux C, Raaijmakers J, Martinotti MG, Pierrat J-C, Garbaye J. 2005. Ectomycorrhizal symbiosis affects functional diversity of rhizosphere fluorescent pseudomonads. New Phytol 165:317–328. doi:10.1111/j.1469-8137.2004.01212.x 15720643

[4] Viollet A, Corberand T, Mougel C, Robin A, Lemanceau P, Mazurier S. 2011. Fluorescent pseudomonads harboring type III secretion genes are enriched in the mycorrhizosphere of Medicago truncatula. FEMS Microbiol Ecol 75:457–467. doi:10.1111/j.1574-6941.2010.01021.x 21204867

[5] Nicolitch O, Feucherolles M, Churin JL, Fauchery L, Turpault MP, Uroz S. 2019. A microcosm approach highlights the response of soil mineral weathering bacterial communities to an increase of K and Mg availability. Sci Rep 9:14403. doi:10.1038/s41598-019-50730-y 31591410 PMC6779897

[6] Wu J, Liu S, Zhang H, Chen S, Si J, Liu L, Wang Y, Tan S, Du Y, Jin Z, Xie J, Zhang D. 2025. Flavones enrich rhizosphere Pseudomonas to enhance nitrogen utilization and secondary root growth in Populus. Nat Commun 16:1461. doi:10.1038/s41467-025-56226-w 39920117 PMC11805958

[7] Chiniquy D, Barnes EM, Zhou J, Hartman K, Li X, Sheflin A, Pella A, Marsh E, Prenni J, Deutschbauer AM, Schachtman DP, Tringe SG. 2021. Microbial community field surveys reveal abundant Pseudomonas population in sorghum rhizosphere composed of many closely related phylotypes. Front Microbiol 12:598180. doi:10.3389/fmicb.2021.598180 33767674 PMC7985074

[8] Dias ACF, Dini-Andreote F, Hannula SE, Andreote FD, Pereira e Silva M de C, Salles JF, de Boer W, van Veen J, van Elsas JD. 2013. Different selective effects on rhizosphere bacteria exerted by genetically modified versus conventional potato lines. PLoS One 8:e67948. doi:10.1371/journal.pone.0067948 23844136 PMC3700926

[9] Bergsma-Vlami M, Prins ME, Raaijmakers JM. 2005. Influence of plant species on population dynamics, genotypic diversity and antibiotic production in the rhizosphere by indigenous Pseudomonas spp. FEMS Microbiol Ecol 52:59–69. doi:10.1016/j.femsec.2004.10.007 16329893

[10] Frey-Klett P, Pierrat JC, Garbaye J. 1997. Location and survival of mycorrhiza helper pseudomonas fluorescens during establishment of ectomycorrhizal symbiosis between laccaria bicolor and douglas Fir. Appl Environ Microbiol 63:139–144. doi:10.1128/aem.63.1.139-144.1997 16535478 PMC1389093

[11] Zhang S, Liu X, Zhou L, Deng L, Zhao W, Liu Y, Ding W. 2022. Alleviating soil acidification could increase disease suppression of bacterial wilt by recruiting potentially beneficial rhizobacteria. Microbiol Spectr 10:e02333-21. doi:10.1128/spectrum.02333-21 35254141 PMC9045175

[12] Uroz S, Calvaruso C, Turpault MP, Pierrat JC, Mustin C, Frey-Klett P. 2007. Effect of the mycorrhizosphere on the genotypic and metabolic diversity of the bacterial communities involved in mineral weathering in a forest soil. Appl Environ Microbiol 73:3019–3027. doi:10.1128/AEM.00121-07 17351101 PMC1892860

[13] Uroz S, Buée M, Murat C, Frey‐Klett P, Martin F. 2010. Pyrosequencing reveals a contrasted bacterial diversity between oak rhizosphere and surrounding soil. Environ Microbiol Rep 2:281–288. doi:10.1111/j.1758-2229.2009.00117.x 23766079

[14] Colin Y, Nicolitch O, Turpault MP, Uroz S. 2017. Mineral types and tree species determine the functional and taxonomic structures of forest soil bacterial communities. Appl Environ Microbiol 83:e02684–16. doi:10.1128/AEM.02684-16 28003192 PMC5311413

[15] Gulati A, Rahi P, Vyas P. 2008. Characterization of phosphate-solubilizing fluorescent pseudomonads from the rhizosphere of seabuckthorn growing in the cold deserts of himalayas. Curr Microbiol 56:73–79. doi:10.1007/s00284-007-9042-3 17909886

[16] Browne P, Rice O, Miller SH, Burke J, Dowling DN, Morrissey JP, O’Gara F. 2009. Superior inorganic phosphate solubilization is linked to phylogeny within the Pseudomonas fluorescens complex. Agric, Ecosyst Environ, Appl Soil Ecol 43:131–138. doi:10.1016/j.apsoil.2009.06.010

[17] Calvaruso C, Turpault MP, Leclerc E, Frey-Klett P. 2007. Impact of ectomycorrhizosphere on the functional diversity of soil bacterial and fungal communities from a forest stand in relation to nutrient mobilization processes. Microb Ecol 54:567–577. doi:10.1007/s00248-007-9260-z 17546519

[18] Uroz S, Courty PE, Pierrat JC, Peter M, Buée M, Turpault MP, Garbaye J, Frey-Klett P. 2013. Functional profiling and distribution of the forest soil bacterial communities along the soil mycorrhizosphere continuum. Microb Ecol 66:404–415. doi:10.1007/s00248-013-0199-y 23455431

[19] Pospiech A, Neumann B. 1995. A versatile quick-prep of genomic DNA from gram-positive bacteria. Trends Genet 11:217–218. doi:10.1016/s0168-9525(00)89052-6 7638902

[20] Joshi NA, Fass JN. 2011. Sickle: a sliding-window, adaptive, quality-based trimming tool for FastQ files. https://github.com/najoshi/sickle.

[21] Andrews S. 2010. FastQC: a quality control tool for high throughput sequence data. http://www.bioinformatics.babraham.ac.uk/projects/fastqc.

[22] Wick RR, Judd LM, Gorrie CL, Holt KE. 2017. Unicycler: resolving bacterial genome assemblies from short and long sequencing reads. PLoS Comput Biol 13:e1005595. doi:10.1371/journal.pcbi.1005595 28594827 PMC5481147

[23] Li H. 2018. Minimap2: pairwise alignment for nucleotide sequences. Bioinformatics 34:3094–3100. doi:10.1093/bioinformatics/bty191 29750242 PMC6137996

[24] Li H, Handsaker B, Wysoker A, Fennell T, Ruan J, Homer N, Marth G, Abecasis G, Durbin R, 1000 Genome Project Data Processing Subgroup. 2009. The sequence alignment/map format and SAMtools. Bioinformatics 25:2078–2079. doi:10.1093/bioinformatics/btp352 19505943 PMC2723002

[25] Robinson JT, Thorvaldsdóttir H, Winckler W, Guttman M, Lander ES, Getz G, Mesirov JP. 2011. Integrative genomics viewer. Nat Biotechnol 29:24–26. doi:10.1038/nbt.1754 21221095 PMC3346182

[26] Tegenfeldt F, Kuznetsov D, Manni M, Berkeley M, Zdobnov EM, Kriventseva EV. 2025. OrthoDB and BUSCO update: annotation of orthologs with wider sampling of genomes. Nucleic Acids Res 53:D516–D522. doi:10.1093/nar/gkae987 39535043 PMC11701741

[27] Vallenet D, Calteau A, Dubois M, Amours P, Bazin A, Beuvin M, Burlot L, Bussell X, Fouteau S, Gautreau G, Lajus A, Langlois J, Planel R, Roche D, Rollin J, Rouy Z, Sabatet V, Médigue C. 2020. MicroScope: an integrated platform for the annotation and exploration of microbial gene functions through genomic, pangenomic and metabolic comparative analysis. Nucleic Acids Res 48:D579–D589. doi:10.1093/nar/gkz926 31647104 PMC7145621

[28] Chan PP, Lin BY, Mak AJ, Lowe TM. 2021. tRNAscan-SE 2.0: improved detection and functional classification of transfer RNA genes. Nucleic Acids Res 49:9077–9096. doi:10.1093/nar/gkab688 34417604 PMC8450103

[29] Torsten S. 2026. Barrnap. Available from: https://github.com/tseemann/barrnap

[30] Meier-Kolthoff JP, Göker M. 2019. TYGS is an automated high-throughput platform for state-of-the-art genome-based taxonomy. Nat Commun 10:2182. doi:10.1038/s41467-019-10210-3 31097708 PMC6522516

[31] Švec P, Kosina M, Zeman M, Holochová P, Králová S, Němcová E, Micenková L, Urvashi S, Gupta V, Sood U, Lal R, Korpole S, Sedláček I. 2020. Pseudomonas karstica sp. nov. and Pseudomonas spelaei sp. nov., isolated from calcite moonmilk deposits from caves. Int J Syst Evol Microbiol 70:5131–5140. doi:10.1099/ijsem.0.004393 32821035

[32] Uroz S, Picard L, Turpault MP. 2022. Recent progress in understanding the ecology and molecular genetics of soil mineral weathering bacteria. Trends Microbiol 30:882–897. doi:10.1016/j.tim.2022.01.019 35181182

[33] Blin K, Shaw S, Kloosterman AM, Charlop-Powers Z, van Wezel GP, Medema MH, Weber T. 2021. antiSMASH 6.0: improving cluster detection and comparison capabilities. Nucleic Acids Res 49:W29–W35. doi:10.1093/nar/gkab335 33978755 PMC8262755

